# Effectiveness of a Mobile App Intervention for Anxiety and Depression Symptoms in University Students: Randomized Controlled Trial

**DOI:** 10.2196/15418

**Published:** 2020-07-31

**Authors:** Tayla McCloud, Rebecca Jones, Gemma Lewis, Vaughan Bell, Elias Tsakanikos

**Affiliations:** 1 Division of Psychiatry University College London London United Kingdom; 2 Division of Psychology & Language Sciences University College London London United Kingdom; 3 Health Service and Population Research Department Institute of Psychiatry, Psychology & Neuroscience King’s College London London United Kingdom

**Keywords:** anxiety, depression, cognitive behavioral therapy, eHealth, online intervention, mobile apps, randomized controlled trial, mobile phone

## Abstract

**Background:**

Depression and anxiety symptoms are common among university students, but many do not receive treatment. This is often because of lack of availability, reluctance to seek help, and not meeting the diagnostic criteria required to access services. Internet-based interventions, including smartphone apps, can overcome these issues. However, a large number of apps are available, each with little evidence of their effectiveness.

**Objective:**

This study aims to evaluate for the first time the effectiveness of a self-guided mobile app, Feel Stress Free, for the treatment of depression and anxiety symptoms in students.

**Methods:**

A web-based randomized controlled trial compared a cognitive behavioral therapy (CBT)–based mobile app Feel Stress Free with a wait-list control. University students self-identified as experiencing symptoms of anxiety or depression and were randomized to 6 weeks of intervention (n=84) or control (n=84), unblinded. The app is self-guided and incorporates behavioral relaxation activities, mood tracking and thought challenging, and minigames. Participants completed the Hospital Anxiety and Depression Scale online at baseline and every fortnight.

**Results:**

At week 6, the primary end point, there was evidence that the Feel Stress Free app reduced depression symptoms (mean difference −1.56; 95% CI −2.67 to −0.44; *P*=.006) but only very weak evidence that it reduced anxiety symptoms (mean difference −1.36; 95% CI −2.93 to 0.21; *P*=.09). At week 4, there was evidence to support the effectiveness of the intervention for anxiety symptoms (mean difference −1.94; 95% CI −3.11 to −0.77; *P*=.001) and, though weaker, depression symptoms (mean difference −1.08; 95% CI −2.12 to −0.04; *P*=.04). At week 6, 83% (34/41) of participants indicated that they were using the app weekly or more frequently.

**Conclusions:**

The Feel Stress Free app is a promising mobile intervention for treating symptoms of anxiety and depression in students and overcomes many of the barriers to traditional CBT. Further research is needed to establish its effectiveness at and beyond 6 weeks.

**Trial Registration:**

ClinicalTrials.gov NCT03032952; https://clinicaltrials.gov/ct2/show/NCT03032952

## Introduction

Depression and anxiety are common and disabling disorders and often co-occur [1]. Around 25% of people with depression or anxiety experience symptoms before the age of 20 years [2], and rates are high among university students relative to other sections of the population [3]. A systematic review of international studies estimated the prevalence of depression to be 30.6% among university students [4]. The prevalence of depression is also increasing: a recent report indicated that from 2006 and 2007 to 2015 and 2016, the number of higher education students in the United Kingdom who disclosed mental health disorders to their institution rose five-fold, and university deaths by suicide increased by 79% [3]. Early intervention could prevent adverse outcomes often associated with anxiety and depression, such as substance misuse, educational underachievement, and suicide [5,6]. However, for university students, there can be numerous barriers to help-seeking, including lack of time, privacy concerns, financial constraints, a lack of perceived need for formal help, and stigma [7]. Innovative approaches are therefore needed to address the high burden of mental health problems among university students.

The most established psychological treatment for depression and anxiety, cognitive behavioral therapy (CBT), often has long waiting lists in the United Kingdom, and services are not evenly spread throughout the country [8]. Students who would benefit from timely CBT may also not meet the criteria required to access services, particularly if their symptoms appear to be mild (though still distressing). University mental health services are struggling with increasing demand [3], and a collective of executive heads representing 136 universities in the United Kingdom (Universities UK) has called for higher education leaders to prioritize student mental health care as imperative [9]. In the absence of professional help, self-help approaches have been shown to be a somewhat effective alternative that are highly valued by young people, particularly when in a digital form [10,11].

There is evidence that computerized forms of self-directed CBT (cCBT) can be as effective as traditional CBT in the treatment of depression and anxiety [10,12-14], and cCBT is now recommended in the United Kingdom for treating subclinical to moderate depression [15]. Mobile CBT apps used primarily on smartphones represent an opportunity to distribute cCBT to the 2.6 billion active smartphone users worldwide [16], at a low cost to the provider and the user. Among those aged between 18 and 24 years in the United Kingdom, it was recently estimated that 93% owned or had ready access to a smartphone [17], and on average over 4 hours per day is spent using them [18]. This eliminates the need for therapist input, formal help-seeking, or a clinical diagnosis, and the lack of waiting time has been shown to be particularly attractive among a UK student sample [19].

Promising findings have been reported regarding the effectiveness of app-based interventions for depression and anxiety in student populations. In 2014, a systematic review by Davies et al [20] suggested that internet- and computer-based interventions could be beneficial in improving depression and anxiety, particularly as an adjunct to university support services. Indeed, internet-based self-help is often recommended by university counseling services struggling to cope with high demand [21]. More recently, a 2019 systematic review [22] on digital health interventions for improving depression and anxiety among students found that mobile-based interventions such as apps appear to be as promising as computer-, web-, and virtual reality–based interventions. However, the authors found a comparative scarcity of research in this area: only 8 out of 71 included studies tested interventions delivered via mobile phones. The issue remains that many mental health apps are available, but there is little or no evidence of the effectiveness of the vast majority of these apps [23-26].

The aim of this study (ClinicalTrials.gov registration NCT03032952) was to examine for the first time the effectiveness of a particular CBT-based mobile app, *Feel Stress Free*, as a treatment for symptoms of anxiety and depression among university students.

## Methods

### Design

This was a 6-week, web-based, parallel group, unblinded randomized controlled trial, with a wait-list control. Participants were individually randomized in a 1:1 ratio.

### Participants and Setting

Eligible participants were aged 18 years or over; scored 8 or above on one or both subscales of the Hospital Anxiety and Depression Scale (HADS), indicating at least a possible case of depression and/or anxiety [27]; were currently a student at 1 of the 4 partnered universities; had access to an Apple or Android phone or tablet or a computer with Firefox, Safari, or Chrome installed; and were computer and internet literate.

A total of 4 universities that partnered with Thrive Therapeutic Software Limited agreed to take part: University College London (UCL), School of Oriental and African Studies University of London, University of Buckingham, and University of Roehampton. Students were recruited between March and June 2016 through their university student union or student welfare services via email, poster and social media advertisements, and university welfare staff recommendations. The recruited participants were directed to the Thrive website, where they could enroll by entering their university email address. Participant IDs were then provided via an email to this address, with a link to the web-based information sheet and consent form. Participants were always contacted via their university email to prevent multiple sign-ups. Participants were not compensated in any way for their participation. All data were collected online.

Signing the consent form was the only time during the trial when the participants had to give their names; they were contacted via email and identified using participant IDs only from this point onward. Participants answered a series of questions confirming that they had read the information about the trial and understood what would be asked of them. If a participant answered *No* to any of the questions, a representative of the trial emailed them to clarify any queries and ensured that their informed consent was given before continuing. Next, participants were sent the web-based baseline questionnaires via email, which contained demographic questions and the HADS, which was also used to determine eligibility.

### Sample Size

Sample size was estimated using both subscales of the self-rated HADS as coprimary outcome measures, with the aim of detecting an intervention effect of half an SD. As the estimated SD differs for each subscale, we chose the most conservative value and calculated that at least 64 participants were needed in each arm, with a 2-tailed significance level of .05 and 80% power. Owing to the high dropout rates usually observed in web-based trials [28], it was decided that up to 300 participants would be randomized.

### Randomization and Allocation

Participants were individually randomized in batches of 30 each time this number of students had been screened and confirmed as eligible. Random numbers were generated by a statistician (RJ) to allocate participants within each batch to the 2 study arms in a 1:1 ratio, using prespecified code written in Stata (StataCorp; version 14) [29]. The list of participant IDs and group allocations was then returned to the researcher (TM). At the end of the recruitment period, a final batch of 18 participants was randomized.

The researcher (TM) then emailed a link to download the *Feel Stress Free* app to those allocated to the intervention group. As participants were required to sign up for an account to use the app, it was possible to monitor the participants who had downloaded the app, and reminders were sent to those who had not yet done so. Those allocated to the wait-list group were sent an email informing them that they would receive access to the app at the end of the trial. Day 1 of the trial was defined as the date of randomization for each batch of participants. Owing to the nature of a wait-list control group, we could not blind the researcher or participants to group allocation. However, apart from the allocation email, all participants received exactly the same emails regardless of group, and trial staff had no other way of influencing the participants.

### Intervention

**Figure 1:**
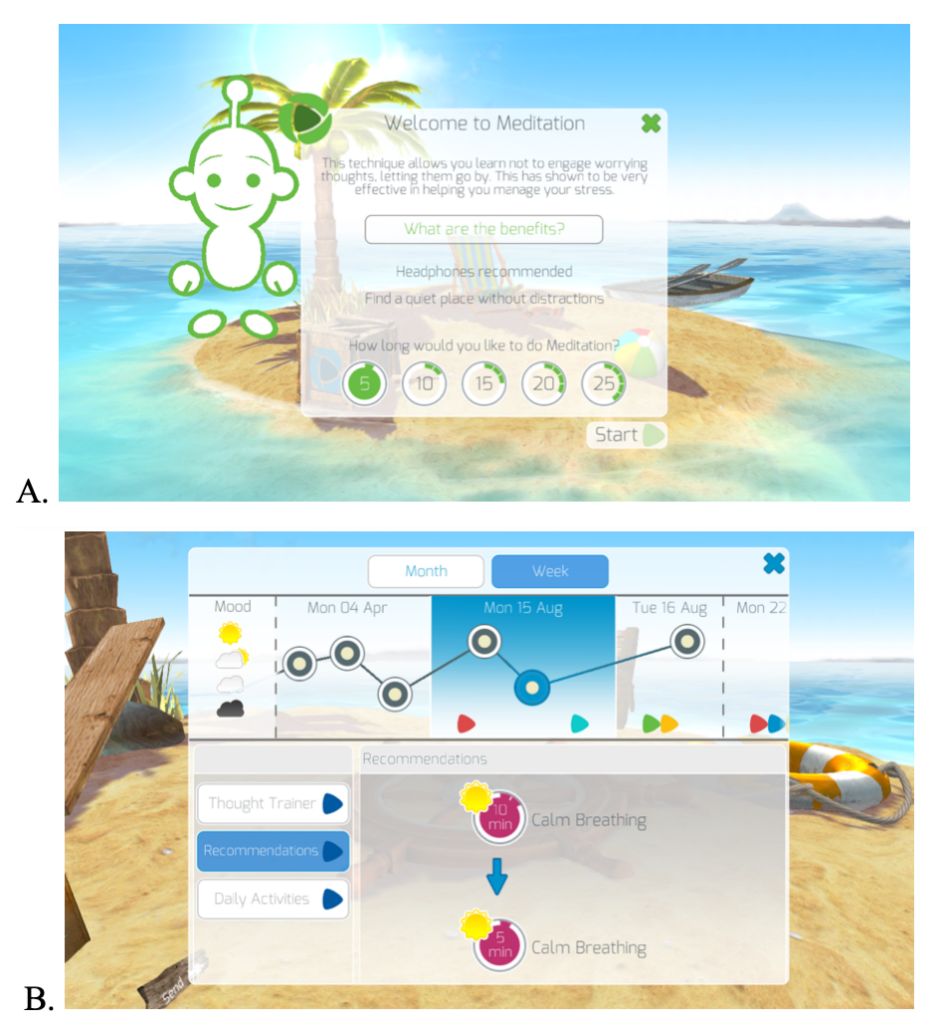
Screenshots of the Feel Stress Free app on a smartphone, showing the landing page for the meditation activity (A) and the mood tracker with an activity recommendation (B).

The *Feel Stress Free* app (version 1.5; Figure 1), developed by Thrive Therapeutic Software Limited, uses CBT-based activities to help users manage symptoms of depression and anxiety. The app comprises 4 behavioral relaxation activities—calm breathing, mindfulness-style meditation, deep muscle relaxation, and self-hypnosis; one cognitive activity, incorporating both mood tracking and thought challenging; a relaxing minigame; and a feature for positive messages in a bottle. *Feel Stress Free* is self-guided (fully automated, with no additional human involvement), and individuals are led around the app by a friendly robot character that makes activity recommendations. Each activity has several options for duration and a short audiovisual guide explaining its use and benefits. Participants were instructed to use the app at least once per week, spending at least 10 min on one or more of the main activities, throughout the trial. There were no prompts or reminders to use the app. Further details of the app and its basis can be found in Multimedia Appendix 1. A very similar version of this app (version 1.3) was also tested as a treatment for agoraphobia in 2017 [30]. More information can be found at https://thrive.uk.com/ [31].

*Feel Stress Free* can be used on any Apple or Android smartphone or tablet or any computer with Firefox, Safari, or Chrome installed. Feel Stress Free is available on the web, although it is primarily a mobile app. Users must be connected to the internet. Participants randomized to receive the intervention were able to download and access the app and all its features free of charge. Those in the wait-list group were able to do so at the end of the study. The app was offered to participants exactly as it is publicly available. No app usage data were available to study researchers, as per Thrive’s privacy policy. Participants in both groups were not limited in the additional care they could receive throughout the trial but were asked at baseline whether they were receiving any concurrent treatment (medication or psychological interventions) for anxiety or depression.

### Measures

The HADS [27] is an established measure of the severity of anxiety and depression symptoms and has been validated for online use in a student population [32]. There are 7 items scored from 0 to 3 on each subscale, giving a possible range of 0 to 21 for each; a score between 8 and 10 inclusive indicates a possible case, and a score of 11 or above indicates a probable case [27]. Specificities and sensitivities are usually reported to be 80% or higher in UK-based research [33]. A previous study did not reveal any differences between online and pen-and-paper versions of the scale [32]. The internal consistency (Cronbach alpha) of the HADS in this study was .68 and .77 at baseline for the anxiety and depression subscales, respectively, and .81 and .86, respectively, at week 6.

The joint primary outcomes were depression and anxiety symptom severity at week 6, as measured by the 2 subscales of the HADS (the HADS-Anxiety Subscale, HADS-A, for anxiety symptoms and the HADS-Depression Subscale, HADS-D, for depression symptoms). Secondary outcomes were HADS-A and HADS-D scores as repeated measures at baseline (screening), week 2, and week 4. The link to complete the web-based questionnaires was sent to all participants via email on the first day of each of the relevant weeks, followed by prompts throughout the week.

Participants were asked how often they had been using the app in the past fortnight as a measure of treatment adherence (*not at all*, *fortnightly*, *weekly*, *a few times*, *several times a week*, *once a day*, *more than once a day*, or *I am not in the app group*). A response indicating weekly or more frequent usage was considered to indicate adherence. Participants also had the opportunity to indicate whether they had experienced any adverse events in the past 2 weeks. If a participant indicated that they had experienced an adverse event, details of the event and its severity (on a scale of 1-3, with 1 being mild and 3 being severe) were requested within the questionnaire.

### Statistical Analyses

All main analyses were intention-to-treat (ITT) analyses. The primary outcomes (HADS-D and HADS-A scores at 6 weeks) were analyzed using linear mixed models (a separate model for each outcome), with the scores from each time point treated as a repeated measures outcome. Models were adjusted for age, gender, and concurrent treatment, as these variables were expected to be strongly associated with the outcome. An interaction between each covariate and time permitted the effect of the covariates to differ at each time point. We first report the effect on the primary outcomes (HADS-D and HADS-A scores at week 6) and then on the secondary outcomes. A random effect of participant with an unstructured residual covariance matrix allowed for correlations between these repeated measures on individuals over time. Fixed effects of treatment (intervention vs wait-list group), time (baseline and 2, 4, and 6 weeks follow-up), and the interaction between treatment and time were specified. The estimated baseline score was constrained to be identical in the 2 study arms, equivalent to adjusting for baseline and permitting the relationship between the baseline and follow-up scores to differ at each time point. Standardized effect sizes (ESs) were produced by performing similar analyses with outcome variables standardized by the mean and SD of the whole sample at baseline. A per-protocol analysis was also undertaken to examine the effectiveness of the intervention under ideal conditions. This analysis compared all participants in the wait-list group with only those participants in the intervention group who had indicated treatment adherence at all 3 time points, using statistical models similar to those of the main trial analysis. All analyses were conducted using Stata version 14 (StataCorp) [29].

### Ethical Approval

This research was approved by the UCL Ethics Committee (reference number 8227/001). All participants gave informed consent via a web-based form before participating, and adverse events were monitored at each time point. Data were anonymized, stored, and protected according to the UK Data Protection Act (2018) and the General Data Protection Regulation guidelines (2018). The trial was registered and reported in accordance with the Consolidated Standards of Reporting Trials of Electronic and Mobile Health Applications and Online Telehealth (CONSORT-EHEALTH) checklist [34]. Protocol changes can be found in Multimedia Appendix 2.

## Results

### Participants

**Figure 2:**
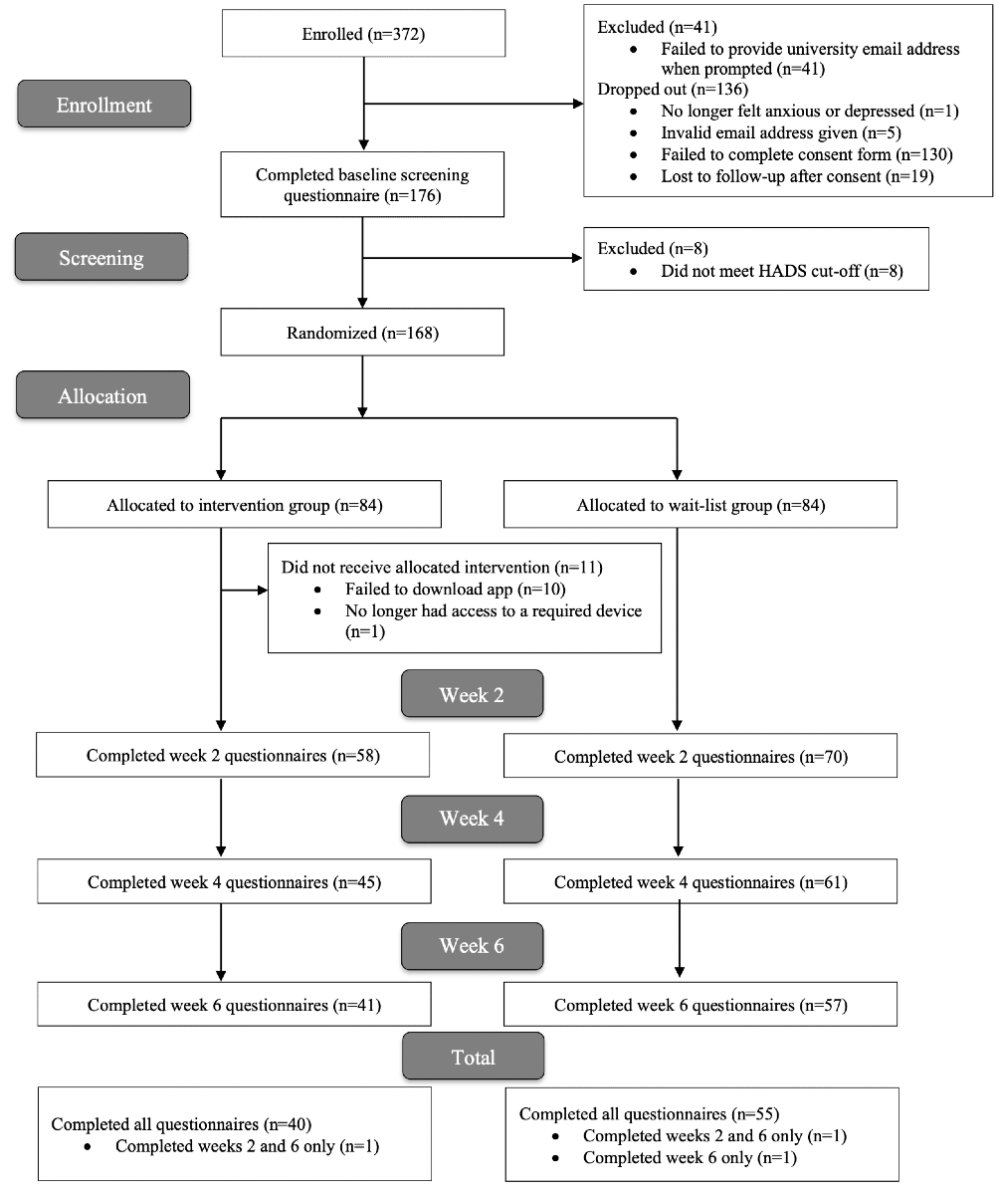
Flow of participants through the trial.

During the recruitment period, 372 students enrolled in the trial; 195 out of 372 (52.4%) completed the consent form. The baseline questionnaires were sent to these consenting participants, and 176 of the 195 participants (90.3%) completed them, 8 of whom were ineligible. The remaining 168 participants were randomized—84 to each study arm. Figure 2 shows the flow of participants through the trial.

In the intervention group, one participant was unable to download the app owing to an unexpected lack of access to a device. A further 10 participants did not download the app. All participants were sent questionnaires at all 3 follow-up time points, regardless of whether they had completed the previous questionnaires. All participants were included in the main analyses in the groups to which they were randomized.

### Baseline Characteristics

The mean age of all 168 participants was 24.3 years (SD 6.71; range 18-54 years), 82.7% (139/168) of the participants were female, and 61.9% (104/168) were undergraduate students. At baseline, the mean score on HADS-A was 13.7 (SD 3.33) and on HADS-D was 8.31 (SD 3.96). At baseline, 45% (75/168) of the participants reported that they would have an exam or a dissertation deadline during the trial period, and 25.0% (42/168) reported that they were receiving another treatment concurrently. These characteristics were well balanced across groups, apart from concurrent treatment; more participants in the intervention group were receiving other forms of treatment during the study than in the wait-list control group (30% and 20%, respectively). Baseline demographic and clinical information for all randomized participants are presented in Table 1.

**Table 1:** Baseline demographic and clinical characteristics by study arm.

Characteristics			Intervention group (n=84)		Wait-list group (n=84)
Age (years), mean (SD)			25.1 (7.68)		23.5 (5.53)
**Gender, n (%)**					
	Male	13 (15)		10 (12)	
	Female	69 (82)		74 (88)	
	Prefer not to say	2 (2)		0 (0)	
**University, n (%)**					
	University College London	37 (44)		37 (44)	
	University of Roehampton	34 (40)		35 (42)	
	School of Oriental and African Studies, University of London	8 (10)		7 (8)	
	University of Buckingham	5 (6)		5 (6)	
**Graduate status, n (%)**					
	Undergraduate	51 (61)		53 (63)	
	Postgraduate	33 (39)		31 (37)	
Assessment during trial, n (%)			38 (45)		37 (44)
Concurrent treatment, n (%)			25 (30)		17 (20)
**Participants with HADS^a^ score >8^b^, n (%)**					
	HADS-Anxiety Subscale (HADS-A)	83 (99)		82 (98)	
	HADS-Depression Subscale (HADS-D)	46 (55)		45 (54)	
	Comorbid depression and anxiety	45 (54)		43 (51)	

^a^HADS: Hospital Anxiety and Depression Scale.

^b^A Hospital Anxiety and Depression Scale (HADS) subscale score of more than 8 indicates possible depression or anxiety. Participants who met this criterion on both subscales of the HADS are additionally indicated as having comorbid depression and anxiety, respectively.

### Main Analyses

**Figure 3:**
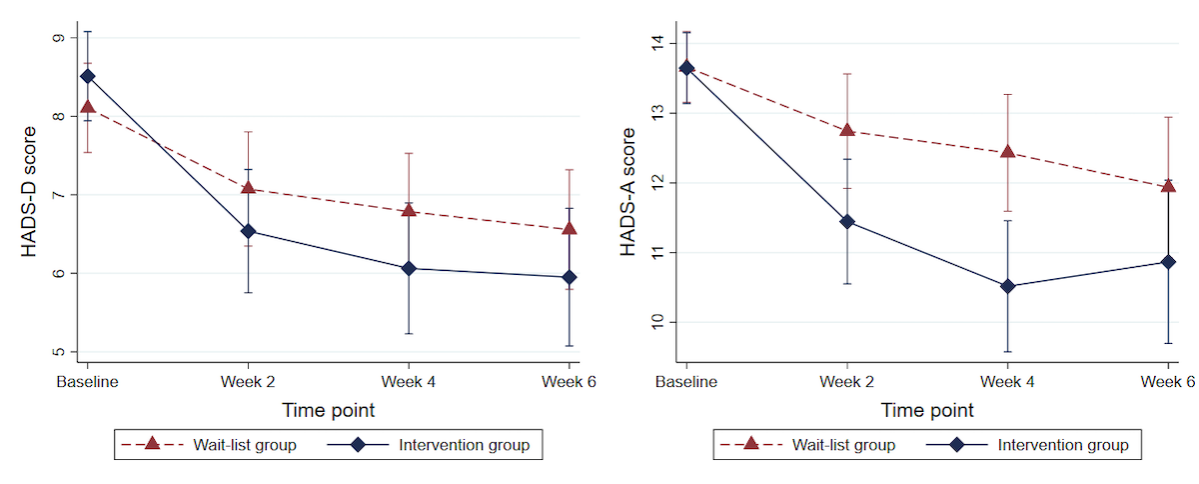
Hospital Anxiety and Depression Scale anxiety and depression scores by study arm, estimated by the adjusted multilevel model at each time point. Error bars represent 95% CIs. HADS-A: Hospital Anxiety and Depression Scale-Anxiety Subscale; HADS-D: Hospital Anxiety and Depression Scale-Depression Subscale.

The mean scores for each HADS subscale by study arm and time point are shown in Table 2. We found evidence that the *Feel Stress Free* app reduced depression at 6 weeks follow-up (adjusted mean difference [MD] −1.56; 95% CI −2.67 to −0.44; *P*=.006; standardized ES=0.39); but only very weak evidence was found of a reduction in anxiety (adjusted MD −1.36; 95% CI −2.93 to 0.21; *P*=.09). At week 4, there was evidence of the effectiveness of the app in reducing symptoms of both depression (adjusted MD −1.08; 95% CI −2.12 to −0.04; *P*=.04; ES=0.27) and anxiety (adjusted MD −1.94; 95% CI −3.11 to −0.77; *P*=.001; ES=0.58). There was weak evidence for an effect of the intervention on anxiety symptoms at week 2, but no evidence of a treatment effect for depression symptoms. These results were consistent with the unadjusted model, with the exception of week 2, where there was weak evidence of the app’s effectiveness in reducing both anxiety and depression symptoms in the unadjusted model. The multilevel model results are presented in Table 3. Figure 3 shows the HADS anxiety and depression subscale scores by study arm, estimated by model 2. A sensitivity analysis adjusting additionally for university can be found in Multimedia Appendix 3 (first table).

**Table 2:** Hospital Anxiety and Depression Scale anxiety and depression scores by study arm at baseline and at 2, 4, and 6 weeks of follow-up.

Scale and time point		Intervention group		Wait-list group		
		n	Mean (SD)		n	Mean (SD)
**HADS^a^-Anxiety Subscale**						
	Week 6	41	10.8 (4.25)		57	11.8 (4.60)
	Week 4	45	10.1 (3.85)		61	12.1 (4.19)
	Week 2	58	11.2 (3.75)		70	12.6 (3.88)
	Baseline	84	13.4 (3.25)		84	13.9 (3.41)
**HADS-Depression Subscale**						
	Week 6	41	5.8 (3.72)		57	6.6 (4.07)
	Week 4	45	5.9 (3.63)		61	6.6 (3.61)
	Week 2	58	6.3 (3.20)		70	6.9 (3.84)
	Baseline	84	8.3 (3.73)		84	8.3 (4.20)

^a^HADS: Hospital Anxiety and Depression Scale.

**Table 3:** Estimated effect of Feel Stress Free intervention on Hospital Anxiety and Depression Scale anxiety and depression scores at 2, 4, and 6 weeks of follow-up.

Timepoint			Model 1^a^						Model 2^a^					
			Estimate (95% CI)		*P* value		Effect size^b^		Estimate (95% CI)		*P* value		Effect size^b^	
**HADS^c^-Anxiety Subscale**														
	**Primary**													
		Week 6		−1.08 (−2.62 to 0.47)		.17		0.32		−1.36 (−2.93 to 0.21)		.09		0.41
	**Secondary**													
		Week 4		−1.94 (−3.06 to −0.82)		.001		0.58		−1.94 (−3.11 to −0.77)		.001		0.58
		Week 2		−1.27 (−2.39 to −0.15)		.03		0.38		−1.10 (−2.28 to 0.07)		.07		0.33
**HADS-Depression Subscale**														
	**Primary**													
		Week 6		−1.26 (−2.37 to −0.16)		.03		0.32		−1.56 (−2.67 to −0.44)		.006		0.39
	**Secondary**													
		Week 4		−1.20 (−2.21 to −0.19)		.02		0.30		−1.08 (−2.12 to −0.04)		.04		0.27
		Week 2		−0.98 (−1.91 to −0.06)		.04		0.25		−0.67 (−1.62 to 0.27)		.16		0.17

^a^Estimates are from linear mixed models, with scores from each time point treated as a repeated measures outcome. In both models, the baseline score was constrained to be identical in the 2 study arms, equivalent to adjusting for baseline. Model 2 adjusted additionally for age, gender, and presence of concurrent treatment.

^b^Effect size standardized by mean and SD of sample at baseline.

^c^HADS: Hospital Anxiety and Depression Scale.

### Attrition and Adherence

Overall, 58.3% (98/168) of the randomized participants completed the week 6 questionnaires. A larger proportion of participants in the wait-list group provided outcome data at week 6 (57/84, 68%) than in the intervention group (41/84, 49%; χ^2^_1_=6.3; *P*=.01). We used univariable logistic regression models to identify baseline variables associated with having incomplete outcome data (missing questionnaire data at any time point). Postgraduates were less likely to have missing outcome data than undergraduates (odds ratio 0.44; 95% CI 0.23 to 0.84; *P*=.01), but there were no differences in terms of any other baseline characteristics. Therefore, we conducted a sensitivity analysis adjusting for graduate status to investigate the possible effect of missing outcome data. The treatment effect was similar. More details can be found in Multimedia Appendix 3 (second table).

Adherence to the treatment was defined as using the app weekly or more frequently. By this definition, 98% of the participants reported adherence at week 2, 89% at week 4, and 83% at week 6 (Table 4). Of the participants who completed all the follow-up questionnaires, 80% (32/40) indicated adherence at every time point.

**Table 4:** Usage data for the intervention group at each time point. Adherence was defined as using the app weekly or more frequently, measured via self-report.

Usage	Week 2 (n=56), n (%)	Week 4 (n=45), n (%)	Week 6 (n=41), n (%)
Less than weekly	1 (2)	5 (11)	7 (17)
Weekly	9 (16)	10 (22)	6 (15)
A few times a week	28 (48)	19 (42)	20 (49)
Several times a week	17 (29)	8 (18)	5 (12)
Daily or more	3 (5)	3 (7)	3 (7)

### Per-Protocol Analysis

We performed a prespecified per-protocol analysis including only those in the intervention group who indicated treatment adherence at every time point (32/84, 38%) and all participants in the wait-list control group (n=84). Although no longer an unbiased sample, the estimated adjusted treatment effects at the primary end point (week 6) were similar to those from the ITT analysis for both anxiety symptoms (adjusted MD −1.37; 95% CI −3.17 to 0.42; *P*=.13) and depression symptoms (adjusted MD −1.88; 95% CI −3.12 to −0.64; *P*=.003). At week 4, there was also evidence to support the effectiveness of the app at reducing both anxiety (adjusted MD −2.11; 95% CI −3.50 to −0.73, *P*=.003) and depression symptoms (adjusted MD −1.13; 95% CI −2.35 to 0.08; *P*=.07), again in line with the ITT analysis.

### Safety

Two participants in the app group reported adverse events associated with the intervention. One participant reported at week 4 that they were “feeling reliant on it” and “recognising deeper thoughts and emotions” and rated the severity of these unpleasant effects as mild. The second participant reported that they experienced feelings of stress and anxiety when they had technical difficulties with the app, which they reported as being of moderate severity at week 2 and of mild severity at week 6. There were no other reports of any adverse events experienced as a result of the intervention.

## Discussion

### Principal Findings

We found preliminary evidence that the *Feel Stress Free* app reduced depression and anxiety symptoms after 6 weeks. At secondary time points, we observed greater reductions in depression and anxiety symptoms in the intervention group compared with the control group after 4 weeks. Treatment adherence and usage of the app were encouraging (83% reported using the app weekly or more frequently at week 6), and very few adverse events were reported. These results provide preliminary support for the effectiveness of this mobile CBT-based app in treating depression and anxiety symptoms in a student population.

The standardized ESs for statistically significant comparisons were 0.39 (week 6) and 0.27 (week 4) for depression, which are considered small [35], and 0.58 (week 4) for anxiety, which are considered medium [35]. These are in line with other recent studies that compare CBT apps with wait-list or inactive controls; meta-analyses by Firth et al reported standardized mean difference ESs of 0.45 for anxiety [36] and 0.56 for depression [37]. Although these meta-analyses reported smaller ESs for studies comparing similar apps with active controls (0.19 and 0.22 for anxiety and depression, respectively), for students on a waiting list or for whom therapist-supported CBT or face-to-face CBT is not an option, a readily available intervention that produces even a relatively small effect after 4 weeks would be of value.

Although a minimum clinically important difference (MCID) has not been established for the HADS in the general population, the best estimate we have is from trials of those with chronic pulmonary obstructive disorder. Although MCIDs are a complex issue likely to be best represented by percentage rather than absolute score changes [38], in these trials, a change in score of 1.5 points is generally accepted as the MCID [39,40]. Our adjusted mean difference estimates slightly exceeded this value at week 6 for depression but was below it at week 4. For anxiety, the adjusted mean difference estimate was below this value (and not statistically significant) at week 6 but exceeded this value at week 4. On the basis of 95% CIs for these estimates, we cannot rule out the potential for a clinically significant effect of this intervention, but we also cannot rule out the values in the lower range, which would be small and not clinically important. Overall, these findings should be treated with caution regarding clinical importance.

### Strengths

This study avoided many methodological issues common throughout the internet-based CBT literature. In particular, adherence to the app-based treatment was clearly defined and measured and not just assumed from the completion of outcome measures. This allowed us to make observations regarding app usage separate from study participation—for instance, postgraduates were less likely to have missing data than undergraduates, but not more likely to indicate treatment adherence, as may have been assumed otherwise. We can, therefore, report the important positive outcome that 83% of those in the intervention group continued to use the app regularly at week 6.

Offering the app to users as it would be in a real-world setting and without supervision allowed the study to have a higher level of external validity than most trials. In addition, the ITT approach to analysis provides a more valid estimate of the effectiveness of the intervention, by reflecting the protocol deviations and noncompliance common in clinical practice. Participants self-administered all outcome measures online and had no personalized contact with trial personnel, which means that observer bias can be discounted.

This study contributes new data to the body of literature surrounding cCBT and more specifically to the emerging field of mobile- and app-based CBT. Our findings suggest that mobile CBT without therapist support or a structured session-based approach may improve symptoms of anxiety and depression in a sample with a range of symptom severities, including subclinical and severe. This study also provides preliminary evidence for the effectiveness of the attributes of this particular app, which can be directly compared with other apps of this kind to consider the ideal characteristics of cCBT and internet-based CBT to optimize effectiveness.

### Limitations

This study has several limitations. One issue was attrition, as only 58% of the participants completed the week 6 questionnaires. However, this rate is comparable with what is seen at similar time points in other trials of mobile- and web-based CBT where therapist contact is minimal or absent [28,41,42]. Furthermore, although the sample size at week 6 was relatively small, the multilevel modeling analysis technique used allows all participants to contribute to analyses, even those with missing follow-up data, which increases the statistical power. Nevertheless, future research should focus on trying to ascertain the reasons behind dropouts, particularly in relation to app design. In this study, it is unclear whether those in the intervention group who stopped completing questionnaires were doing so because of the questionnaires or because they did not want or need to use the app any more. Fewer participants dropped out in the wait-list group (Table 2), which may be because they remained interested in the trial as a way to access the app. Overall, the attrition observed appears to be realistic for a web-based trial comparing a CBT-based app with a wait-list control group [42]. Future web-based trials could try to incorporate some elements of interaction with participants to improve retention, for example, an in-person consent procedure and demonstration of the app and regular reminders to use the app.

It must be considered that our estimate of adherence was based on self-reported data subject to social desirability bias, and the true rate of adherence may be lower. However, if we did overestimate adherence, the fact that low levels of usage produced a benefit would provide support for the effectiveness of the app even without frequent use, in line with findings by Firth et al [36]. Nevertheless, more detailed data on participants’ usage of the app, potentially including qualitative feedback on its perceived usefulness and user experience, would be beneficial to the field. Future studies should incorporate direct monitoring of the participants’ app usage where possible to ascertain whether there is a dose-response relationship and whether usage of certain activities within the app may be associated with better outcomes and/or preferred by participants [43].

A further limitation of this study is the use of a wait-list control group. As it was not possible to blind participants, the groups differed in their expectation of improvement, and a placebo effect in the intervention group could have inflated ESs or made a type I error more likely. In particular, a so-called *digital placebo* has been suggested by some researchers, in that the observed benefits could arise from the increased use of the electronic device itself [44]. Nevertheless, this intervention requires such little investment on the part of distributors and patients alike that this would not discredit its use entirely, particularly for those who are waiting for another treatment. In this way, our comparison between a mobile CBT-based app and a control group lacking in expectation of improvement can be considered to reflect the real-world situation of those at whom this intervention is aimed and should be considered in this context of increased external validity. In addition, the wait-list group also showed improvement during the trial, thus reducing the differences between groups. This is common in trials and likely because of spontaneous improvement; fluctuations in symptoms; and regression to the mean, all of which accompany the passage of time [45]. However, we cannot rule out the possibility that the control group sought out and used other CBT-based mental health apps or other treatments during the course of the trial, thus diminishing the observed effect of the *Feel Stress Free* app.

Our sample was 85% female, which could limit the generalizability of our results. Although we had more female students taking part than expected, some gender imbalance is common in trials and likely to partly reflect gender differences in prevalence and help-seeking behaviors. Further research could investigate whether female students are more interested in mobile app interventions than male students, and apps could be then tailored accordingly. The results of this trial may also not be generalizable beyond university students, but arguably, these are the individuals who are most likely to benefit from the accessibility of this type of treatment [46]. The sample was a mix of those with subclinical and clinical symptoms to reflect this population. The promising results indicate the possible effectiveness of the app in potentially treating and preventing clinical levels of symptoms.

### Future Directions

Further research is needed to confirm the effectiveness of this mobile intervention at and beyond 6 weeks, particularly for anxiety. Our results are in line with the oft-reported finding that the majority of the improvement associated with a CBT app (and traditional CBT [47]) is seen in the short term [48,49], but a longer follow-up period will be needed to ascertain whether our week 6 results represent a fluctuation typically seen around the 6-week time point. To rule out the digital placebo effect as an explanation for our results, this app should also be compared with an active, smartphone-based control. As more evidence is gathered for individual CBT apps, comparisons between different apps are warranted, followed by a consideration of the characteristics that the most effective apps have in common—a recent meta-analysis attempted this but was not able to draw robust conclusions [37].

This app could be offered to university students, particularly where the demand for therapies exceeds the provision. The app could be offered to those who are on the waiting list for traditional CBT; it may be particularly useful when a rapid improvement is imperative, such as before exams. Trying self-directed forms of CBT may also make individuals more likely to seek traditional CBT [50] and can be an alternative for those who are unable to attend therapy. Students are unlikely to have a problem accessing a device on which the app can be used, and benefits were observed regardless of age and gender. Internet-based interventions such as *Feel Stress Free* would also fit well within a stepped care model, such as that currently in place in the United Kingdom; it could be offered during the period of guided self-help [15,,51], followed by traditional CBT if this is not beneficial. Future research could also explore the use of internet-based interventions as an adjunct to traditional CBT, for use between sessions.

### Conclusions

We found preliminary evidence to support the use of *Feel Stress Free*, a CBT-based mobile app, as a short-term intervention for students experiencing symptoms of depression and anxiety. Further research is needed to establish the potential benefit of the app, in particular by comparing it with an active control in a larger sample less vulnerable to dropouts.

## Appendix

Multimedia Appendix 1 Feel Stress Free app activities, rationale and screenshots.

Multimedia Appendix 2 Protocol changes.

Multimedia Appendix 3 Sensitivity analyses.

Multimedia Appendix 4 CONSORT EHEALTH form V1.6.

## Abbreviations

CBT: cognitive behavioral therapy
cCBT: computerized forms of self-directed cognitive behavioral therapy
ES: effect size
HADS: Hospital Anxiety and Depression Scale
HADS-A: Hospital Anxiety and Depression Scale-Anxiety Subscale
HADS-D: Hospital Anxiety and Depression Scale-Depression Subscale
ITT: intention-to-treat
MCID: minimum clinically important difference
UCL: University College London

## References

[1] Kessler RC, Berglund P, Demler O, Jin R, Koretz D, Merikangas KR, Rush AJ, Walters EE, Wang PS, National Comorbidity Survey Replication (2003). The epidemiology of major depressive disorder: results from the national comorbidity survey replication (NCS-R). J Am Med Assoc.

[2] Kessler RC, Angermeyer M, Anthony JC, de Graaf R, Demyttenaere K, Gasquet I, de Girolamo G, Gluzman S, Gureje O, Haro JM, Kawakami N, Karam A, Levinson D, Medina Mora ME, Browne MA, Posada-Villa J, Stein DJ, Tsang CH, Aguilar-Gaxiola S, Alonso J, Lee S, Heeringa S, Pennell BE, Berglund P, Gruber MJ, Petukhova M, Chatterji S, Ustün TB (2007). Lifetime prevalence and age-of-onset distributions of mental disorders in the world health organization's world mental health survey initiative. World Psychiatry.

[3] Thorley C (2017). Not By Degrees: Improving Student Mental Health in the UK's Universities. IPPR.

[4] Ibrahim AK, Kelly SJ, Adams CE, Glazebrook C (2013). A systematic review of studies of depression prevalence in university students. J Psychiatr Res.

[5] Lewinsohn PM, Hops H, Roberts RE, Seeley JR, Andrews JA (1993). Adolescent psychopathology: I. Prevalence and incidence of depression and other DSM-III-R disorders in high school students. J Abnorm Psychol.

[6] Woodward LJ, Fergusson DM (2001). Life course outcomes of young people with anxiety disorders in adolescence. J Am Acad Child Adolesc Psychiatry.

[7] Hunt J, Eisenberg D (2010). Mental health problems and help-seeking behavior among college students. J Adolesc Health.

[8] Cavanagh K (2014). Geographic inequity in the availability of cognitive behavioural therapy in England and Wales: a 10-year update. Behav Cogn Psychother.

[9] #stepchange: Mental Health in Higher Education. Universities UK.

[10] Kaltenthaler E, Brazier J, de Nigris E, Tumur I, Ferriter M, Beverley C, Parry G, Rooney G, Sutcliffe P (2006). Computerised cognitive behaviour therapy for depression and anxiety update: a systematic review and economic evaluation. Health Technol Assess.

[11] Gulliver A, Griffiths KM, Christensen H (2010). Perceived barriers and facilitators to mental health help-seeking in young people: a systematic review. BMC Psychiatry.

[12] Spence SH, Donovan CL, March S, Gamble A, Anderson RE, Prosser S, Kenardy J (2011). A randomized controlled trial of online versus clinic-based CBT for adolescent anxiety. J Consult Clin Psychol.

[13] Ly KH, Trüschel A, Jarl L, Magnusson S, Windahl T, Johansson R, Carlbring P, Andersson G (2014). Behavioural activation versus mindfulness-based guided self-help treatment administered through a smartphone application: a randomised controlled trial. BMJ Open.

[14] Carlbring P, Andersson G, Cuijpers P, Riper H, Hedman-Lagerlöf E (2018). Internet-based vs face-to-face cognitive behavior therapy for psychiatric and somatic disorders: an updated systematic review and meta-analysis. Cogn Behav Ther.

[15] (2009). Depression in Adults: Recognition and Management (CG90). The National Institute for Health and Care Excellence (NICE).

[16] (2016). Mobile Internet: Smartphones for Everyone?. Nakono — UK Consumer Product Reviews & Market Research.

[17] (2019). Global Mobile Consumer Survey: UK Cut. Deloitte.

[18] (2019). UK Digital Market Overview Report Q3 2019. UK Online Measurement (UKOM).

[19] Musiat P, Goldstone P, Tarrier N (2014). Understanding the acceptability of e-mental health--attitudes and expectations towards computerised self-help treatments for mental health problems. BMC Psychiatry.

[20] Davies EB, Morriss R, Glazebrook C (2014). Computer-delivered and web-based interventions to improve depression, anxiety, and psychological well-being of university students: a systematic review and meta-analysis. J Med Internet Res.

[21] Kitzrow M (2003). The mental health needs of today's college students: challenges and recommendations. J Stud Aff Res Pract.

[22] Lattie EG, Adkins EC, Winquist N, Stiles-Shields C, Wafford QE, Graham AK (2019). Digital mental health interventions for depression, anxiety, and enhancement of psychological well-being among college students: systematic review. J Med Internet Res.

[23] Chan S, Torous J, Hinton L, Yellowlees P (2015). Towards a framework for evaluating mobile mental health apps. Telemed J E Health.

[24] Anthes E (2016). Mental health: there's an app for that. Nature.

[25] Stoll R, Pina A, Gary K, Amresh A (2017). Usability of a smartphone application to support the prevention and early intervention of anxiety in youth. Cogn Behav Pract.

[26] (2018). Evidence Standards Framework for Digital Health Technologies. The National Institute for Health and Care Excellence (NICE).

[27] Zigmond AS, Snaith RP (1983). The hospital anxiety and depression scale. Acta Psychiatr Scand.

[28] Christensen H, Griffiths KM, Farrer L (2009). Adherence in internet interventions for anxiety and depression. J Med Internet Res.

[29] StataCorp M, Banks J (2015). tata Statistical Software: Release 14.

[30] Christoforou M, Fonseca JA, Tsakanikos E (2017). Two novel cognitive behavioral therapy-based mobile apps for agoraphobia: randomized controlled trial. J Med Internet Res.

[31] Herrmann C (1997). International experiences with the hospital anxiety and depression scale--a review of validation data and clinical results. J Psychosom Res.

[32] Whitehead L (2011). Methodological issues in Internet-mediated research: a randomized comparison of internet versus mailed questionnaires. J Med Internet Res.

[33] Herrmann C (1997). International experiences with the hospital anxiety and depression scale--a review of validation data and clinical results. J Psychosom Res.

[34] Eysenbach G, CONSORT-EHEALTH Group (2011). CONSORT-EHEALTH: improving and standardizing evaluation reports of web-based and mobile health interventions. J Med Internet Res.

[35] Cohen J (1988). Statistical Power Analysis for the Behavioral Sciences. Second Edition.

[36] Firth J, Torous J, Nicholas J, Carney R, Rosenbaum S, Sarris J (2017). Can smartphone mental health interventions reduce symptoms of anxiety? A meta-analysis of randomized controlled trials. J Affect Disord.

[37] Firth J, Torous J, Nicholas J, Carney R, Pratap A, Rosenbaum S, Sarris J (2017). The efficacy of smartphone-based mental health interventions for depressive symptoms: a meta-analysis of randomized controlled trials. World Psychiatry.

[38] Button KS, Kounali D, Thomas L, Wiles NJ, Peters TJ, Welton NJ, Ades AE, Lewis G (2015). Minimal clinically important difference on the beck depression inventory--ii according to the patient's perspective. Psychol Med.

[39] Puhan MA, Frey M, Büchi S, Schünemann HJ (2008). The minimal important difference of the hospital anxiety and depression scale in patients with chronic obstructive pulmonary disease. Health Qual Life Outcomes.

[40] Bhandari NJ, Jain T, Marolda C, ZuWallack RL (2013). Comprehensive pulmonary rehabilitation results in clinically meaningful improvements in anxiety and depression in patients with chronic obstructive pulmonary disease. J Cardiopulm Rehabil Prev.

[41] Clarke G, Reid E, Eubanks D, O'Connor E, DeBar LL, Kelleher C, Lynch F, Nunley S (2002). Overcoming depression on the internet (ODIN): a randomized controlled trial of an internet depression skills intervention program. J Med Internet Res.

[42] Proudfoot J, Clarke J, Birch M, Whitton AE, Parker G, Manicavasagar V, Harrison V, Christensen H, Hadzi-Pavlovic D (2013). Impact of a mobile phone and web program on symptom and functional outcomes for people with mild-to-moderate depression, anxiety and stress: a randomised controlled trial. BMC Psychiatry.

[43] Bakker D, Kazantzis N, Rickwood D, Rickard N (2016). Mental health smartphone apps: review and evidence-based recommendations for future developments. JMIR Ment Health.

[44] Torous J, Firth J (2016). The digital placebo effect: mobile mental health meets clinical psychiatry. Lancet Psychiatry.

[45] Rutherford BR, Mori S, Sneed JR, Pimontel MA, Roose SP (2012). Contribution of spontaneous improvement to placebo response in depression: a meta-analytic review. J Psychiatr Res.

[46] Beattie A, Shaw A, Kaur S, Kessler D (2009). Primary-care patients' expectations and experiences of online cognitive behavioural therapy for depression: a qualitative study. Health Expect.

[47] Ilardi S, Craighead W (1994). The role of nonspecific factors in cognitive-behavior therapy for depression. Clin Psychol Sci Pract.

[48] Warmerdam L, van Straten A, Jongsma J, Twisk J, Cuijpers P (2010). Online cognitive behavioral therapy and problem-solving therapy for depressive symptoms: exploring mechanisms of change. J Behav Ther Exp Psychiatry.

[49] Watts S, Mackenzie A, Thomas C, Griskaitis A, Mewton L, Williams A, Andrews G (2013). CBT for depression: a pilot RCT comparing mobile phone vs computer. BMC Psychiatry.

[50] Christensen H, Griffiths K, Groves C, Korten A (2006). Free range users and one hit wonders: community users of an internet-based cognitive behaviour therapy program. Aust N Z J Psychiatry.

[51] Andrews G, Cuijpers P, Craske MG, McEvoy P, Titov N (2010). Computer therapy for the anxiety and depressive disorders is effective, acceptable and practical health care: a meta-analysis. PLoS One.

