# Supplementary material for: Effectiveness of a Mobile App Intervention for Anxiety and Depression Symptoms in University Students: Randomized Controlled Trial
Source: JMIR Mhealth Uhealth. 2020 Jul 31;8(7):e15418. doi: 10.2196/15418 (PMC7428915; doi:10.2196/15418)
Supplement: Multimedia Appendix 1 [file mhealth_v8i7e15418_app1.docx]

Multimedia Appendix 1 – Feel Stress Free app activities, rationale and screenshots.

Calm breathing encourages the user to control their breathing, taking slow and deep breaths to relax. This activity forms the basis for the remaining three behavioural activities, and can be practised for 3, 5, 7, 10 or 15 minutes.

The meditation activity is based on mindfulness techniques shown to be efficacious in reducing symptoms of depression and anxiety (see Khoury et al. [1]). Users practise rhythmic breathing and are encouraged to take an accepting attitude towards their thoughts, sensations and feelings throughout. Meditations last for 5, 10, 15, 20 or 25 minutes.

Deep muscle relaxation lasts for seven and a half minutes, and involves controlled breathing whilst tensing and releasing muscles progressively throughout the body [2].

The self-hypnosis activity aims to teach the user to go into a hypnotic trance as a state of relaxation [3], and lasts for three minutes excluding the recommended deep muscle relaxation beforehand.

“Mood Meter” allows users to rate how positive their mood is at any time along a visual analogue scale. Users are then offered a few general words to pick from to describe how they feel, for example ‘anxious’ if they have indicated that they are feeling negative, or ‘optimistic’ if they have indicated that they are feeling positive (see Figure 1). They have the option to input their own words and can select several before continuing. If they initially indicated that they felt positive, the activity finishes at this point, but if they indicated they felt negative they continue through to “Thought Trainer”.

“Thought Trainer” prompts the user to identify the situation, triggers and thoughts surrounding their feelings, again with suggestions and options to input their own and select several. Depending on which are chosen, alternative interpretations are suggested based on Ellis’ ABC technique of cognitive restructuring [4]. At the end of the “Thought Trainer” and/or “Mood Meter” activity, the app recommends activities for the user to complete that day, such as three 15-minute meditation sessions. Individuals are not limited in the number of times an activity can be completed, nor prompted to return to the app.

A user can view their mood in a weekly or monthly view at any time. Users can select a day to review the data inputted during the “Thought Trainer” activity on that day, the recommendations given based on this, and the activities that were completed (see Figure 1). Individuals are also able to choose positive messages (“Message in a Bottle”) to send out to other (anonymous) users, and can play a relaxing mini-game (“Zen Garden”), in which the user designs a sand garden as a distraction technique when dealing with acute stress.

**Figure 1.** Screenshots of the “Feel Stress Free” app on a smartphone. These show the landing page for the behavioural activities (A), the landing page for the meditation activity (B), the “Mood Meter” activity (C), and the mood tracker with an activity recommendation (D).


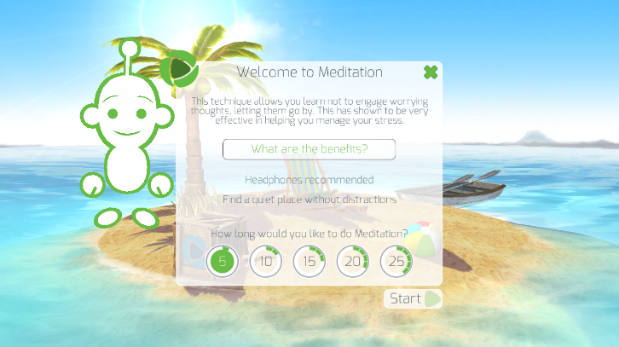

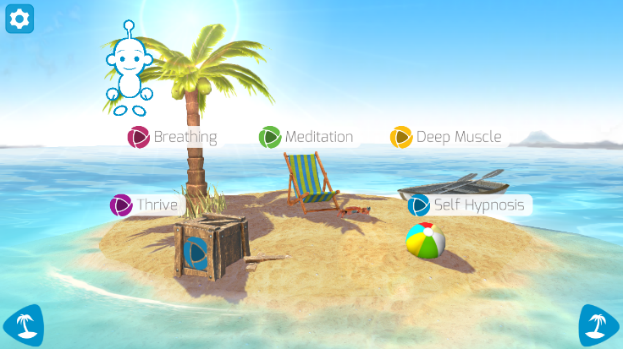


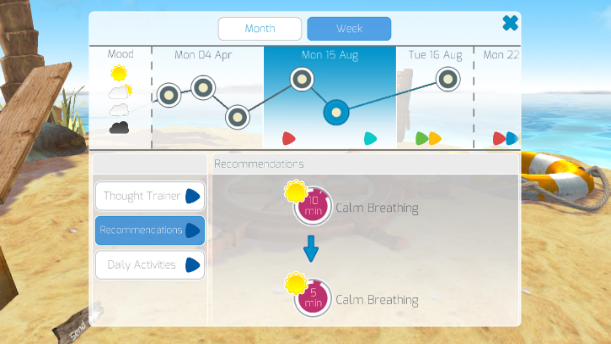

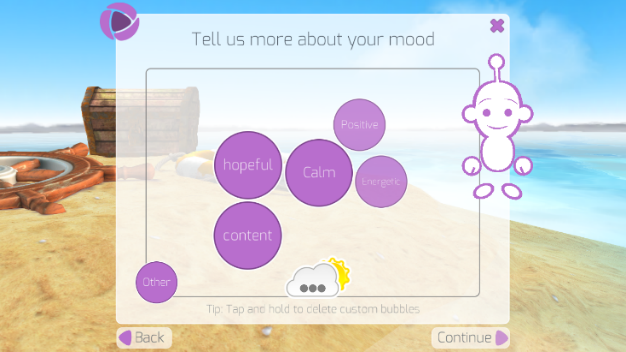


B.

D.

C.

A.

References

1. Khoury B, Lecomte T, Fortin G, Masse M, Therien P, Bouchard V, Chapleau M, Paquin K, Hofmann SG. Mindfulness-based therapy:A comprehensive meta-analysis. Clin Psychol Rev 2013;33(6):763–771. PMID:23796855

2. Conrad A, Roth WT. Muscle relaxation therapy for anxiety disorders: It works but how? J Anxiety Disord 2007;21(3):243–264. PMID:16949248

3. Hammond DC. Hypnosis in the treatment of anxiety- and stress-related disorders. Expert Rev Neurother 2010;10(2):263–273. PMID:20136382

4. Ellis A. The revised ABC’s of rational-emotive therapy (RET). J Ration Cogn Ther 1991;9(3):139–172. [doi: 10.1007/BF01061227]
