# Supplementary material for: Effectiveness of a Mobile App Intervention for Anxiety and Depression Symptoms in University Students: Randomized Controlled Trial
Source: JMIR Mhealth Uhealth. 2020 Jul 31;8(7):e15418. doi: 10.2196/15418 (PMC7428915; doi:10.2196/15418)
Supplement: Multimedia Appendix 2 [file mhealth_v8i7e15418_app2.docx]

Multimedia Appendix 2 – Protocol changes.

- The trial was planned to last for 12 weeks, with the first 6 weeks as the treatment period and the subsequent 6 weeks as a follow-up period. However, due to resource and time constraints and unexpected delays with recruitment, there was substantial attrition and so the follow-up data has not been analysed.

- The secondary outcome of PHQ-9 has not been analysed.
