# Supplementary material for: Effectiveness of a Mobile App Intervention for Anxiety and Depression Symptoms in University Students: Randomized Controlled Trial
Source: JMIR Mhealth Uhealth. 2020 Jul 31;8(7):e15418. doi: 10.2196/15418 (PMC7428915; doi:10.2196/15418)
Supplement: Multimedia Appendix 3 [file mhealth_v8i7e15418_app3.docx]

|  | **Model 2**^a^ | | | | **Model 3**^a^ | | | |
| --- | --- | --- | --- | --- | --- | --- | --- | --- |
|  | Estimate (95% CI) | | P value | | Estimate (95% CI) | P value | |  |
| **HADS Anxiety**^b^ | |  | |  |  |  |  |  |
| *Primary* |  | |  | |  | |  |  |
| Week 6 | -1.36 (-2.93 to 0.21) | | .090 | | -1.41 (-3.02 to 0.19) | | .084 |  |
| *Secondary* |  | |  | |  | |  |  |
| Week 4 | -1.94 (-3.11 to -0.77) | | .001 | | -1.99 (-3.15 to -0.83) | | .001 |  |
| Week 2 | -1.10 (-2.28 to 0.07) | | .065 | | -1.14 (-2.29 to 0.02) | | .054 |  |
| **HADS Depression**^b^ | | | | |  | |  |  |
| *Primary* |  | |  | |  | |  |  |
| Week 6 | -1.56 (-2.67 to -0.44) | | .006 | | -1.69 (-2.80 to -0.57) | | .003 |  |
| *Secondary* |  | |  | |  | |  |  |
| Week 4 | -1.08 (-2.12 to -0.04) | | .042 | | -1.12 (-2.14 to -0.10) | | .031 |  |
| Week 2 | -0.67 (-1.62 to 0.27) | | .162 | | -0.69 (-1.64 to 0.26) | | .157 |  |

Multimedia Appendix 3 – Sensitivity analyses.

**Table 1.** Estimated effect of “Feel Stress Free” intervention on HADS anxiety and depression scores at 2, 4 and 6 weeks follow up. Sensitivity analysis additionally adjusting for university.

^a^Estimates are from linear mixed models with scores from each time point treated as a repeated measures outcome. In both models, baseline score was constrained to be identical in the two study arms, equivalent to adjusting for baseline. Model 2 adjusted for age, gender, and presence of concurrent treatment (as in main manuscript), and Model 3 adjusted additionally for university.

^b^HADS = Hospital Anxiety and Depression Scale.

|  | **Model 2**^a^ | | **Model 4**^a^ | |
| --- | --- | --- | --- | --- |
|  | Estimate (95% CI) | P value | Estimate (95% CI) | P value |
| **HADS Anxiety**^b^ | | | | |
| *Primary* |  |  |  |  |
| Week 6 | -1.36 (-2.93 to 0.21) | .090 | -1.27 (-2.85 to 0.30) | .112 |
| *Secondary* |  |  |  |  |
| Week 4 | -1.94 (-3.11 to -0.77) | .001 | -1.95 (-3.12 to -0.77) | .001 |
| Week 2 | -1.10 (-2.28 to 0.07) | .065 | -1.12 (-2.29 to 0.06) | .062 |
| **HADS Depression**^b^ | | | | |
| *Primary* |  |  |  |  |
| Week 6 | -1.56 (-2.67 to -0.44) | .006 | -1.54 (-2.67 to -0.42) | .007 |
| *Secondary* |  |  |  |  |
| Week 4 | -1.08 (-2.12 to -0.04) | .042 | -1.11 (-2.15 to -0.07) | .036 |
| Week 2 | -0.67 (-1.62 to 0.27) | .162 | -0.69 (-1.64 to 0.25) | .154 |

**Table 2.** Estimated effect of “Feel Stress Free” intervention on HADS anxiety and depression scores at 2, 4 and 6 weeks follow up. Sensitivity analysis additionally adjusting for variables associated with missingness.

^a^Estimates are from linear mixed models with scores from each time point treated as a repeated measures outcome. In both models, baseline score was constrained to be identical in the two study arms, equivalent to adjusting for baseline. Model 2 adjusted for age, gender, and presence of concurrent treatment (as in main manuscript), and Model 3 adjusted additionally for graduate status.

^b^HADS = Hospital Anxiety and Depression Scale.
